# Are you sure? The relationship between response certainty and performance in visual detection using a perimetry-style task

**DOI:** 10.1167/jov.20.8.27

**Published:** 2020-08-26

**Authors:** Phillip Bedggood, Aiza Ahmad, Adam Chen, Rachael Lim, Sadiqa Maqsudi, Andrew Metha

**Affiliations:** Department of Optometry and Vision Sciences, The University of Melbourne, Parkville, Victoria, Australia; Department of Optometry and Vision Sciences, The University of Melbourne, Parkville, Victoria, Australia; Department of Optometry and Vision Sciences, The University of Melbourne, Parkville, Victoria, Australia; Department of Optometry and Vision Sciences, The University of Melbourne, Parkville, Victoria, Australia; Department of Optometry and Vision Sciences, The University of Melbourne, Parkville, Victoria, Australia; Department of Optometry and Vision Sciences, The University of Melbourne, Parkville, Victoria, Australia

**Keywords:** Confidence, certainty, psychometric function, threshold, slope

## Abstract

Conventional psychophysical methods ignore the degree of confidence associated with each response. We compared the psychometric function for detection with that for “absolute certainty” in a perimetry-style task, to explore how knowledge of response certainty might aid the estimation of detection thresholds. Five healthy subjects performed a temporal 2-AFC detection task, indicating on each trial whether they were “absolutely certain.” The method of constant stimuli was used to characterize the shape of the two psychometric functions. Four eccentricities spanning central and peripheral vision were tested. Where possible, conditions approximated those of the Humphrey Field Analyzer (spot size, duration, background luminance, test locations). Based on the empirical data, adaptive runs (ZEST) were simulated to predict the likely improvement in efficiency obtained by collecting certainty information. Compared to detection, threshold for certainty was 0.5 to 1.0 dB worse, and slope was indistinguishable across all eccentricities tested. A simple two-stage model explained the threshold difference; under this model, psychometric functions for detection and for certainty-given-detection are the same. Exploiting this equivalence is predicted to reduce the number of trials required to achieve a given level of accuracy by approximately 30% to 40%. The chances of detecting a spot and the chances of certainty-given-detection were approximately the same in young, healthy subjects. This means, for example, that a spot detected at threshold was labeled as “certainly” detected approximately half the time. The collection of certainty information could be used to improve the efficiency of estimation of detection thresholds.

## Introduction

The gold standard for accurate measurement of psychophysical thresholds is forced-choice testing, which minimizes bias by negating the observer's internal decision criterion. However, forced-choice testing is inefficient; a single correct response in a two-alternative, forced-choice (2-AFC) trial has a 50% chance of simply being a “lucky guess.” The information content in such a response is thus diluted by noise—this is the price paid for mitigating internal response bias. This problem is exacerbated in the popular scheme for adaptive testing in which a uniform prior probability for threshold is adopted, which is also designed to reduce bias. Accordingly, it is typical for adaptive 2-AFC procedures in nonclinical vision research to use around 30 to 40 trials to estimate threshold (King-Smith, Grigsby, Vingrys, Benes, & Supowit, 1994; Kontsevich & Tyler, 1999).

A common alternative to forced-choice testing is for subjects to respond either “Yes” or “No” to having perceived the relevant stimulus characteristic. Although relatively immune to lucky guesses, Yes/No testing is liable to mis-clicks, false-positive and false-negative results. Measured thresholds depend on the subject's internal criteria and are generally elevated because the subject has not been forced to guess. Hence, a 2-AFC approach (Lesmes, Lu, Baek, Tran, Dosher & Albright, 2015) is generally preferred by most groups in basic research applications, despite the requirement for approximately twice as many trials to converge on a stable threshold (King-Smith et al., 1994; Klein, 2001).

In settings that involve a large number of threshold determinations in a limited time frame, forced-choice testing may be impractical. For example, in clinical visual field testing there are many individual locations to test, and it is important to limit patient fatigue; in a widely used implementation each point may receive just two to six presentations (Bengtsson & Heijl, 1998). Accordingly a Yes/No scheme is used rather than a forced-choice approach, in combination with various other “shortcuts,” which may include fewer trials, fewer staircase reversals, or removal of catch trials (Bengtsson & Heijl, 1998; Bengtsson, Olsson, Heijl, & Rootzén, 1997; Heijl et al., 2019). Although these measures save time, they also limit reliability (Phu, Khuu, Agar, & Kalloniatis, 2019) and, hence, reduce statistical power to make a diagnosis, assess treatment efficacy, or detect progression in disease. One solution to this problem is to run more tests or to use more trials per test (Anderson, Bedggood, Kong, Martin, & Vingrys, 2017; Phu, Kalloniatis, Wang, & Khuu, 2019); however, this may increase the burden on the healthcare system and reduce patient compliance. Another solution is to maximize the information gained from each trial (McKendrick, Denniss, & Turpin, 2014; Phu, Kalloniatis, & Khuu, 2018). The present work seeks to achieve this through consideration of response certainty.

Speaking more broadly than visual psychometric testing, across all classes of perception there is a rich complexity to the percept that gets distilled into each decision to give a particular response, which is not typically transmitted to the experimenter. That is, each percept is automatically and inextricably (Kiani & Shadlen, 2009) accompanied by an internal perception of degree, and of the associated *confidence* in the decision that was made to press or not press a particular button (Klein, 2001). Collection and use of such information has potential to significantly improve the efficiency of psychometric testing, allowing more accurate thresholds in a finite number of trials or faster thresholds for a desired level of accuracy. This is all the more true for forced-choice paradigms, especially those adopting a uniform prior, because these are the most likely to repeatedly display stimuli far from the observer's threshold.

Relatively few groups have explored the use of confidence information to improve the efficiency of threshold determination in psychometric testing (Klein, 2001). Most have concentrated on the “unforced choice”, a 2-AFC task with an additional, opt-out, “uncertain” response introduced. Efficiency of this approach has been suggested to rival conventional Yes/No testing, but with reduced bias (García-Pérez & Alcalá-Quintana, 2013; García-Pérez, 2010; Kaernbach, 2001; Watson, Kellogg, Kawanishi, & Lucas, 1973). It has been further proposed that bias may be eliminated by providing a fourth response option (Hsu & Chin, 2014; Klein, 2001; Okamoto, 2012), such that subjects are still forced to choose between the two alternatives but additionally ascribe either a high- or low-confidence rating (i.e., either high- or low-confidence for alternative 1 or 2: four response options). The idea may be extended to a continuous rating of confidence (for example, on a 100-point scale) (Yi & Merfeld, 2016), with the richer information providing even greater efficiency for rapid determination of threshold.

Recent work has extended the addition of an “uncertain” category to Yes/No testing (i.e., Yes/Unsure/No) in an adaptive procedure, again showing rapid convergence (compared with 2-AFC) and returning threshold values that appear criterion-free (compared with Yes/No testing) (Lesmes et al., 2015). However, this approach uses a large number of false-alarm trials that may be impractical where total test time is important.

The work cited above relies on the observer's ability to rate their own confidence, which may or may not be accurate when confidence is low, such as when responding to stimuli near and below threshold (Hsu & Doble, 2015). A more reliable internal percept may be created when an observer is *certain* that they have made a correct decision. From the perspective of mathematical modelling of the psychometric function, certainty (100% confidence) does not exist. In real life, observers often rely on reaching a sufficient degree of confidence so as to become certain of a particular decision (such as deciding when it is safe to cross a busy road).

Here, we evaluate the potential for using response “certainty” to improve the efficiency of threshold determination in vision science. We have chosen to explore a perimetry-style task, since perimetry is a ubiquitous visual psychometric task. In the first section we present empirical data on the shape of the psychometric functions for performance and for certainty in young, healthy subjects. Data were collected contemporaneously for each trial by combining a 2-AFC detection task with a binary “Certain/Uncertain” rating. We hypothesized that each subject would have a particular threshold at which they become “certain” they have seen the stimulus (i.e., 50% chance of being certain), and that this threshold would coincide with a point on the frequency-of-seeing curve well above the performance threshold (for example, the threshold for responding “certain” may equate to perhaps the 80% or 95% chance of detecting the stimulus). In the second section we use the empirically collected data describing the shape of the psychometric functions to simulate a large number of adaptive runs, to predict the likely benefit of incorporating “certain” responses in adaptive testing procedures.

## Methods

### Subjects

Five young, healthy subjects aged 22 to 24 years were recruited from optometry students at the University of Melbourne Department of Optometry and Vision Sciences. Subjects wore their habitual distance correction if applicable (no subjects required a near correction) and had visual acuity of 20/30 or better with this correction.

Subjects supplied written informed consent and the study complied with the tenets of the Declaration of Helsinki. The project was approved by the University of Melbourne Human Ethics Committee.

### Display

A Samsung SyncMaster 2243 BW LCD monitor (Samsung, Seoul, South Korea) operating at 60 Hz was used to display stimuli. The monitor was controlled by an NVIDIA GeForce GTX680 graphics processing unit (NVIDIA, Santa Clara, CA, USA) through a DVI-I connector which allows analog control. A sliding look-up table was used to address the eight-bit display range with nine-bit precision (Metha, Vingrys, & Badcock, 1993). Monitor luminance was calibrated with a PR650 spectro-photometer (Photo Research, Chatsworth, CA, USA).

### Software

Experiments were operated using The Psychophysics Toolbox (Brainard, 1997) version 3, running under MATLAB (Mathworks, Natick, MA, USA). Standard functions in this software were used to display stimuli, record responses, and fit psychometric functions as described below.

### Testing paradigm

**Figure 1.**
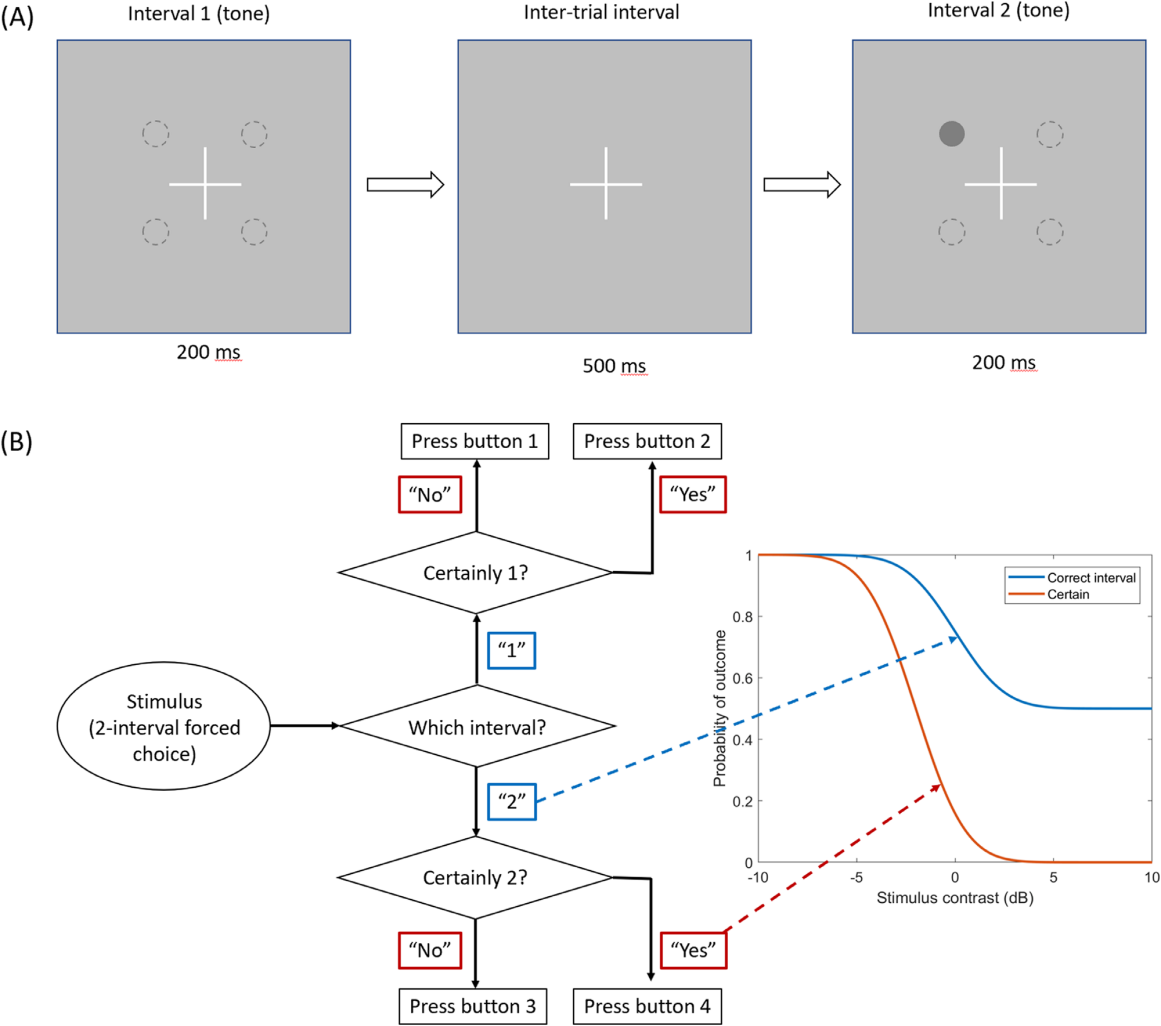
Schematic showing stimulus configuration and response collection. (A) Depicts a temporal 2-AFC task. A Goldmann III spot was displayed for 200 ms in 1 of 4 quadrants, with symmetrically placed co-ordinates (x,y) of ±(3°,3°), (9°,9°), (15°,15°) or (27°,3°) from fixation (*white cross*). The stimulus appeared during either interval 1 or interval 2 (separated by 500 ms). Using a single button press, subjects indicated which interval the spot was in, and whether they were “absolutely certain” of the interval. (B) Depicts the mapping of the observer's percept to button presses and psychometric functions, for an example in which the stimulus was in the second interval and the subject was certain. There were four buttons available, with a button on the subject's left pressed for interval 1 (labels “1” and “2” in the figure) and on the right for interval 2 (labels “3” and “4” in the figure). One row of buttons was used for a noncertain response (labels “1” and “3” in the figure) and a separate row for an “absolutely certain” response (labels “2” and “4”). This allowed contemporaneous measurement of the psychometric functions (*right*) for detection (*blue*) and for certainty (*red*).

Figure 1A depicts the stimulus and task. A gray spot increment (Goldmann III size) was displayed monocularly on a gray background (10 cd/m^2^) for 200 ms, in one of two time intervals separated by 500 ms (temporal 2-AFC). Subjects indicated which interval contained the spot, and whether they were “absolutely certain” regarding their choice of interval (see flow chart in Figure 1B). The 2-AFC paradigm was chosen (as opposed to a Yes/No paradigm) to provide a criterion-free threshold measure for performance, for comparison to the potentially criterion-dependent certainty data.

The method of constant stimuli (MOCS) was used to capture the shape of the psychometric functions. There were seven discrete intensities tested 40 times each. One run, testing a single eccentricity, took approximately 10 minutes. Longer run times were predicted to degrade responses because of fatigue; hence, it was deemed undesirable to test multiple eccentricities in a single run.

During each MOCS run, stimuli were displayed at one of four quadrants positioned symmetrically a fixed distance from fixation (a white cross 1 arc min thick). This spatial uncertainty was introduced to remove any incentive for shifting fixation, given that only a single eccentricity would be tested in each run. Accordingly each block of 40 trials referred to above represents the pooling of data from 10 trials in each of the four quadrants, forming a single block of 40 responses for a given distance from fixation (i.e., responses are averaged across the direction from fixation). Combining spot locations in this way could broaden the slopes measured; to assess this we repeated all analyses with one hemifield removed in either the horizontal or vertical direction and observed that the significance of statistical associations presented in the Results did not change (analysis not shown for brevity).

To decide on the set of stimulus intensities for each subject at a given eccentricity, initial Bayesian testing (ZEST, 40 trials) was conducted to estimate threshold. Based on the fitted psychometric function, 7 test intensities were selected targeting the frequency-of-seeing curve between 2.5% to 97.5% (with equal spacing between intensities in log-contrast space). If the MOCS run subsequently obtained did not reach at least 80% correct at the highest test intensity, a curve was fit to the data and used to suggest a new set of test intensities (again, spanning 2.5% to 97.5% correct). This process was repeated as needed, producing 1 to 3 MOCS runs per subject at each eccentricity. All data were utilized for curve fitting, because even the preliminary runs provide useful information on the asymptotes of the psychometric function.

**Figure 2.**
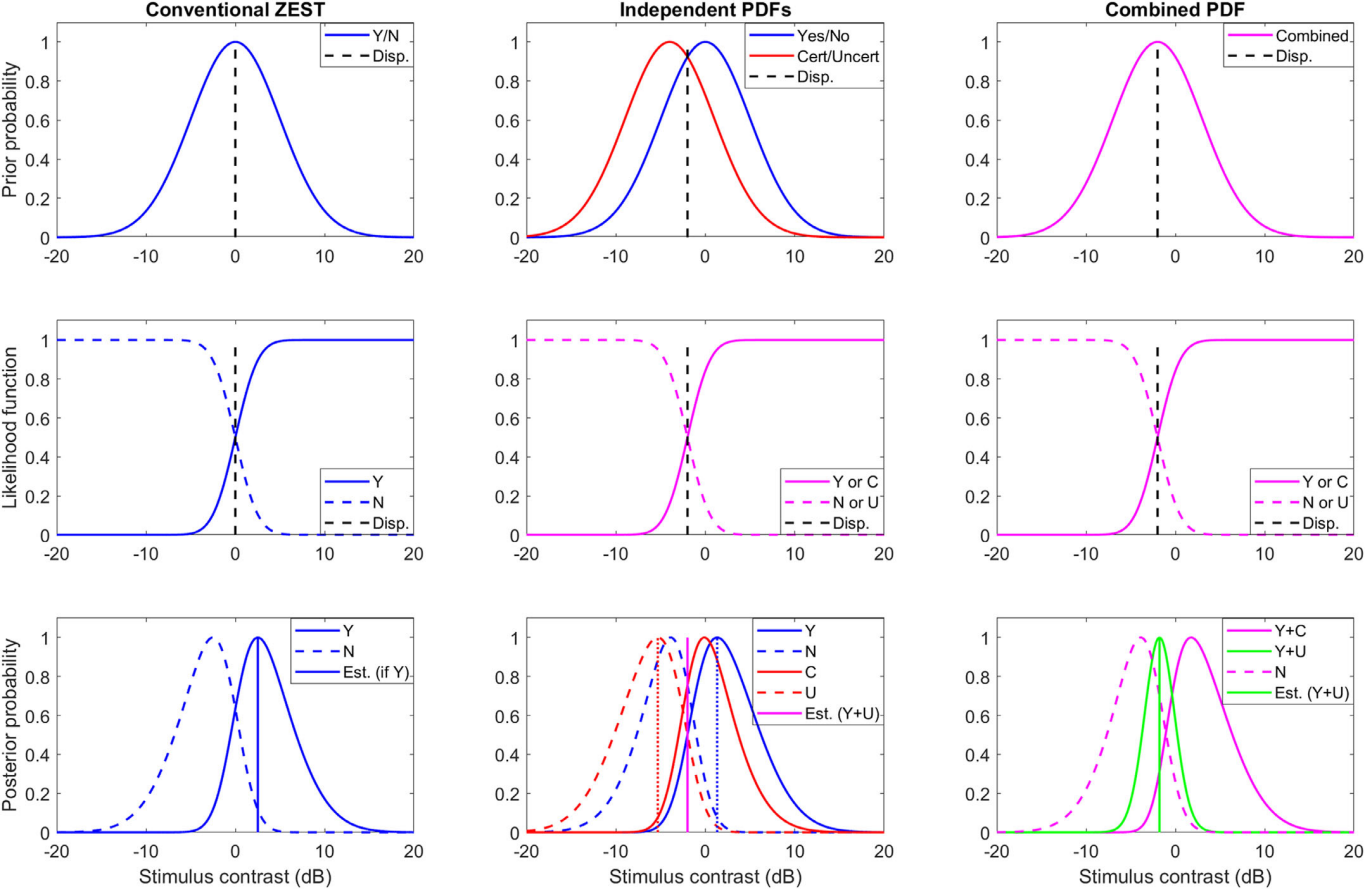
Strategies for combining detection and certainty information in adaptive simulations. *Left-hand column*: conventional Bayesian adaptive testing. *Middle column*: independent probability functions maintained for detection and for certainty, with the estimated threshold being an average of the two. *Right-hand column*: combined probability function maintained for both detection and certainty. *Top row*: example prior probability before a given trial is shown. *Middle row*: example likelihood functions. *Bottom row*: posterior probability after each possible response, together with the new threshold estimate in the case the stimulus was detected, but not with certainty.

**Figure 3.**
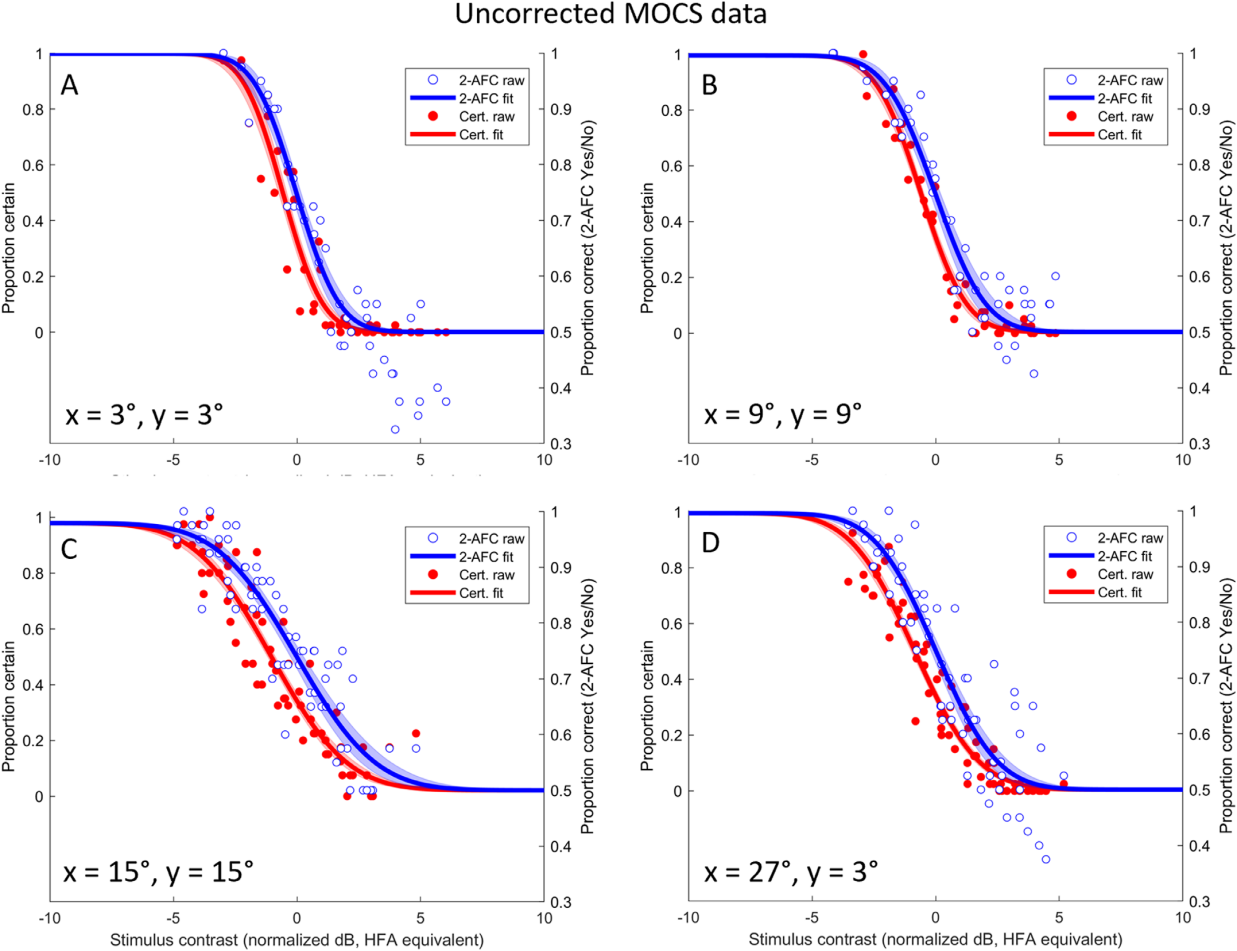
Normalized, pooled MOCS data at four eccentricities. (A–D) show data collected for all subjects from locations corresponding to the following HFA 24-2 points: (A) shows data at 3°,3°; (B) shows data at 9°,9°; (C) shows data at 15°,15°; (D) shows data at 27°,3°. Each datum shows the result from one run (40 trials) in one subject at a given stimulus contrast. Proportion correct (2-AFC) data are plotted in blue against the right-hand y-axes. Raw data are indicated by *open blue symbols*, the curve fit (equally weighted for all plotted points) by the *solid blue line* and ± 1 σ on the curve fit by the *blue shaded region*. Proportion certain (Certain/Not) are plotted in *red* against the left-hand y-axes. Raw data are indicated by *filled red symbols*, the curve fit by the *solid red line* and ± 1 σ on the curve fit by the *red shaded region*. For all panels the threshold for a Certain response was significantly different to that for detection, whereas slope was not distinguishable between the curves (see Table 1).

For the 3°, 3° point, three of five subjects displayed sensitivity for the 200 ms stimuli greater than was allowed by the bit depth of the monitor control (nine bits), so that seven unique MOCS intensities could not be generated. In these subjects the stimulus duration was reduced from 200 ms to 16.7 ms (a single frame), effectively lowering their sensitivity to allow sufficient bit-depth at this location. Results from the other two subjects at this eccentricity were not included for analysis (i.e., Figure 2a and Figure 3a show data from three subjects rather than five).

To facilitate comparison to other work, where possible conditions were chosen to approximate those of the Humphrey Field Analyzer (HFA) (Zeiss, Oberkochen, Germany): the spot size (Goldmann III), stimulus duration (200 ms), background intensity (10 cd/m^2^), testing distance (300 mm), and eccentricities selected (x°–y° from fixation: 3°–3°, 9°–9°, 15°–15°, and 27°–3°) were all chosen in this way. It should be noted that due to the symmetric spot configuration, 2 of the 4 test locations for the 27-3 eccentricity do not correspond to the HFA, which tests this eccentricity only in the nasal visual field. We also used a central fixation target as does the HFA, although our was a white cross subtending 1°, and 1 arc min thick.

### Subject responses

An important feature of the experimental design was the contemporaneous collection of performance (2-AFC) and certainty (Certain/Uncertain) data. Subjects operated a gamepad controller with 4 potential button presses, and were tasked with reporting whether the stimulus was presented in the first or second interval by pressing the left- or right-hand buttons, respectively. Subjects were given unlimited time to respond after each trial. For trials in which the subject felt “absolutely certain” of the correct interval, they were instructed to press the bottom row of buttons on the controller; for all other trials, subjects were to press the top row of buttons. The mapping of subject percepts to button presses and generated psychometric functions is illustrated in Figure 1B. The scheme matches one advocated by Klein (Klein, 2001).

### Curve fitting and statistical analysis for psychophysical response data

The following steps were taken in processing and fitting the data:

1. To correct for interrun and intersubject variability, we first fit the proportion correct (2-AFC) data for each MOCS run to determine threshold. This threshold was subtracted from the test intensities to create a “normalized” threshold of 0 dB (i.e. equivalent to sliding the curve along the x-axis (Strasburger, 2001)). Hence, there are more than seven unique contrast values plotted in the pooled Results figures. The proportion certain data from the same run was shifted by an equal amount, maintaining the relative difference in threshold between the curves.
2. After normalization, at each eccentricity, data were pooled across subjects to maximize statistical power. Each subject contributed between one and three MOCS runs to this pool; all runs were weighted equally for analysis.
3. A fixed lapse rate (response error) was calculated at each eccentricity, by calculating the proportion of trials for which subjects responded with “absolute certainty” but had selected the wrong 2-AFC interval. It is assumed that, in this task, subjects are never “absolutely certain” when no stimulus is presented; this appears justified given that the low lapse rate, averaging 0.8% ± 1% across all subjects and eccentricities. This consideration is returned to in the Discussion in the context of signal detection theory.
4. Pooled 2-AFC and Certain/Uncertain data were each fit with a cumulative normal function. The upper asymptote for both curves was set by (one minus) the lapse rate. The lower asymptote for 2-AFC fits was 0.5, and the lower asymptote for the Certain/Uncertain data was also equal to the lapse rate (following the assumption in the previous step).
5. For statistical significance testing, curve fits were bootstrapped with replacement 10,000 times. Data were bootstrapped by both subject and test intensity to select new combinations of 40-trial response blocks; for each block, the individual responses were also bootstrapped to capture binomial variability.
  • On each resampling a new curve fit was generated; the central 95% of curve-fit parameters determined in this way was used to specify confidence intervals (CI) to test for statistical differences between conditions.
  • To allow visual comparison between whole curves, we also evaluated each bootstrapped curve fit in 0.1 dB increments, ranking the values to produce a 95% confidence interval at each test intensity. These are indicated by the colored patches flanking the solid line curve fits in the results figures. To facilitate comparison between 2-AFC data ranging nominally from 0.5 to 1.0, and proportion certain data ranging nominally from 0.0 to 1.0, the two curves are plotted against independently scaled left- and right y-axes.

### Monte Carlo simulations of Yes/No adaptive testing

Based on the empirical data, we used Monte Carlo simulation to gauge the likely benefit of collecting and using certainty information on each trial of an adaptive procedure. We also explored whether any purported benefit is robust in the presence of common types of error.

In the Results we establish that the psychometric functions for detection and for certainty-given-detection were not distinguishable (to within the limits of our experiment). In other words, we can use a single psychometric function in double-pass to simulate detection and certainty responses: first the function is consulted to decide whether a given stimulus was detected and then, if so, it is consulted again to decide whether it was detected with certainty. For example, if at threshold where we expect 50% of spots to be detected, we expect 50% of 50% = 25% of spots to be detected with certainty. This model assumes that one cannot be “absolutely certain” regarding the presence of a spot which was not detected.

Under this model, we simulated observer responses to a large number of adaptive tests. There were accordingly three possible responses: “no,” “yes, but not with certainty,” and “yes, with certainty.” Depending on the instructions delivered to the subject, this may be equivalent to previously proposed strategies offering subjects a three-category rating system such as “Yes,” ”Uncertain,” and “No” (Lesmes et al., 2015).

Each simulation used Bayesian (ZEST) testing logic. The prior probability distribution for threshold had a mean µ = 0 dB (the initial “guess”), with a relatively uniform distribution (σ = 25.0 dB). The slope parameter was presumed to be 2.0 dB (defining the likelihood functions), which was the midpoint of our parameter range obtained from curve fits to the empirical 2-AFC data (σ = 1.3 dB at 3°,3°; σ = 2.7 dB at 15°,15°). During each adaptive run, the slope parameter remained fixed whereas the current estimate of the threshold parameter was varied.

Simulations were conducted first under “ideal” conditions in which the presumed and actual threshold were equal, presumed and actual slopes were equal, and there were no response errors. Simulations were then repeated under “realistic” sources of error: the presumed threshold (initial guess) was 5 dB from the actual threshold; the slope was 1 dB flatter than expected, and response errors (false positive = false negative) were 5% for both Yes/No and Certain/Uncertain.

For each simulated adaptive run we assessed 3 strategies for selecting the next stimulus intensity, as illustrated in Figure 2.

• “Conventional ZEST”: a typical Yes/No approach (Figure 2, left colum). A single probability density function (PDF) is driven by Yes/No responses using Bayesian inference (example likelihood functions shown in second row of Figure 2). This formed the reference case for the other two strategies.
• “Independent PDFs”: two separate PDFs were maintained (Figure 2, middle column). One PDF incorporates the Yes/No responses (blue) and the other incorportates Certain/Uncertain responses (red). The estimated thresholds according to each of the two PDFs (dashed vertical lines) are averaged (solid magenta line) to estimate threshold.
• “Combined PDF”: a single combined PDF was used (Figure 2, right column)
  ○ For a “No” response, the PDF was multiplied by the appropriate likelihood function in the conventional manner.
  ○ For a “Yes” response, if the response was “Certain” the PDF was multiplied by the appropriate likelihood function *twice*—this is equivalent to having presented two trials at the given intensity, obtaining a “Yes” response for each. This generally produces a greater shift and narrowing of the PDF than would otherwise be the case (solid magenta line in bottom-right panel of Figure 2).
  ○ For a “Yes” response that was not “Certain”, the PDF was multiplied by both the “Yes” *and* the “No” likelihood functions—this is equivalent to having presented two trials at the given intensity, obtaining a “Yes” for one and a “No” for the other. This generally does not shift the PDF, but improves the confidence for the threshold estimate (e.g., the green curve in Figure 2, bottom-right panel is much narrower than other PDFs in the figure).

For each combination of parameters and strategies combined above, we modeled 10,000 runs. This corresponds to a total of 5 (“guess” error levels for threshold) × 3 (“guess” error levels for slope) × 2 (including response errors or not) × 3 (testing strategies) × 10,000 (repeats) = 9 × 10^5^ runs modeled. Each run proceeded for 20 trials, which is suggested to be sufficient for Yes/No testing for threshold estimation (King-Smith et al., 1994).

## Results

### Part I—Experimental data (MOCS)

Figure 3 shows normalized and pooled MOCS data for all five subjects and all four eccentricities, for both proportion correct (2-AFC, right-hand y-axis) and proportion certain (left-hand y-axis). To facilitate visual comparison between curves, shaded regions indicate ±1 standard deviation on the curve fits to the bootstrapped data. For each bootstrap iteration we also calculated the difference in fitted parameters for threshold and slope. At each eccentricity (panels A–D) the fitted threshold for a certain response was less than that for detection; there was no statistically significant difference in fitted slopes (see Table 1). In essence, the certainty curve appears indistinguishable from a detection curve that has been shifted to the left on the order of 0.5 to 1.0 dB.

**Table 1.** Difference in fitted parameters between proportion correct (2-AFC) and proportion certain data. Values indicate 95% confidence intervals for the difference between the fitted parameters. “Slope” refers to the standard deviation of the probability density function (dB), which defines the slope of the psychometric function. At all four eccentricities, thresholds for certainty were significantly different from those for detection, whereas slopes were not significantly different.

	Δ Threshold (dB)			Δ Slope (dB)		
(x,y)	95% CI (lower)	95% CI (upper)	*p* value	95% CI (lower)	95% CI (upper)	*p* value
3°,3°	−0.97	−0.16	**0.006**	−0.59	+0.37	0.64
9°,9°	−1.03	−0.19	**0.000**	−0.75	+0.35	0.56
15°,15°	−1.56	−0.54	**0.000**	−0.82	+0.56	0.83
27°,3°	−1.34	−0.49	**0.000**	−0.50	+0.56	0.72

**Figure 4.**
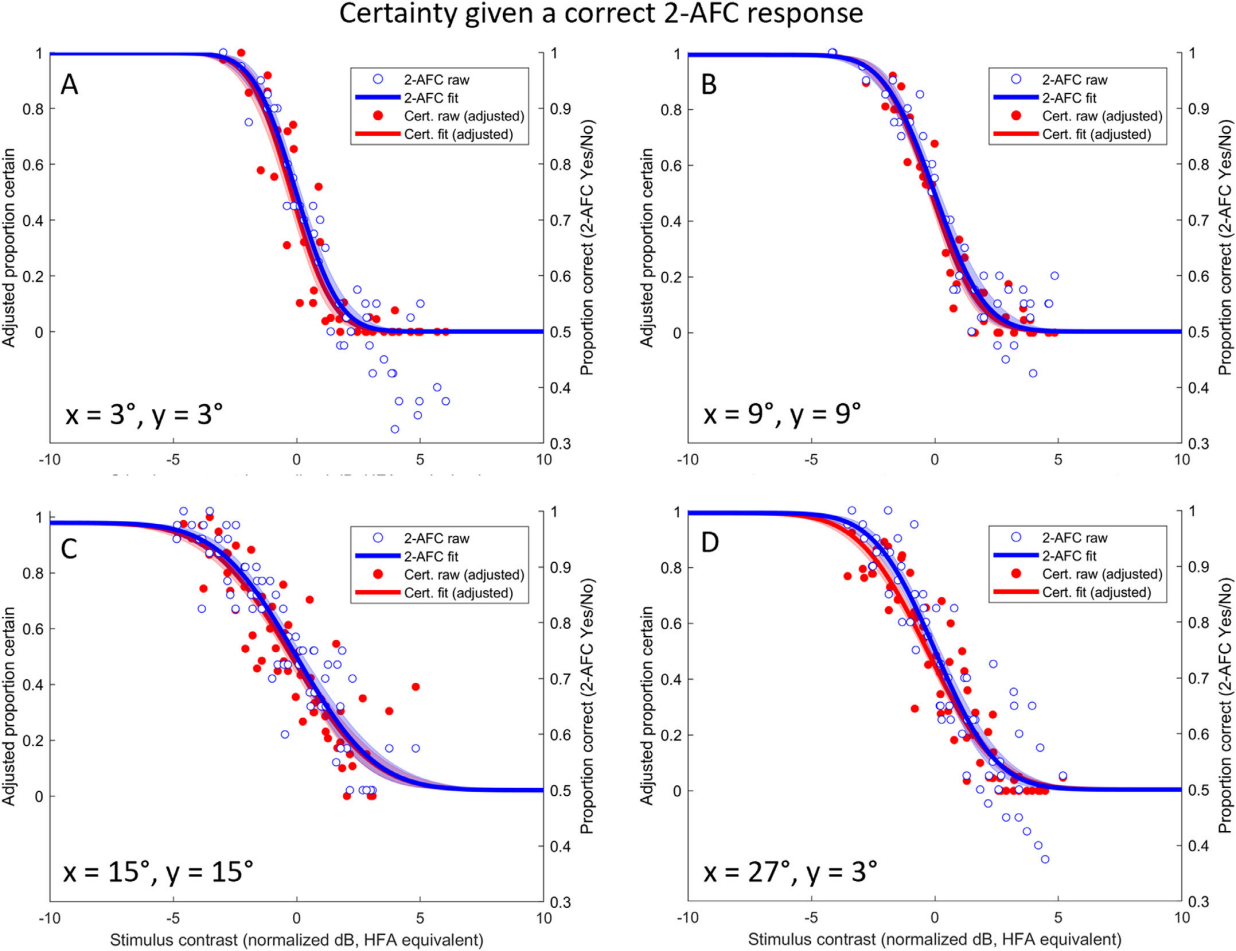
Proportion certain calculated only for those trials in which the subject selected the correct 2-AFC interval. Panels are otherwise identical to Figure 3. For all eccentricities the fitted threshold and slope parameters are now indistinguishable (visual comparison of overlapping (magenta) confidence intervals; also see Table 2).

We next applied a “sanity” correction to the certainty data, reasoning that responses of “absolutely certain” are only obtained where the subject did in fact detect the stimulus. Since it is not possible to know directly which trials were detected in a 2-AFC task, we *partially* corrected for this by considering only those trials in which the observer reported the correct 2-AFC interval. Since some of these must be “lucky guesses” (at threshold, 75% are correct but only 50% should be seen), this will tend to overestimate the proportion of trials detected, and hence underestimate the necessary adjustment to the certainty data. Nonetheless this is an instructive exercise, with results shown in Figure 4 and updated statistical comparisons shown in Table 2. It can be seen that after this transformation the two psychometric functions are not statistically distinguishable in either threshold or slope.

**Table 2.** Difference in fitted parameters between proportion correct (2-AFC) and adjusted certainty data. Values indicate 95% confidence intervals for the difference between the fitted parameters. “Slope” refers to the standard deviation of the probability density function (dB), which defines the slope of the psychometric function. As opposed to Table 1, here the proportion of certain responses was calculated for correct trials only. For all four eccentricities tested, there were no measurable differences in either threshold or slope, meaning that the underlying curves were not distinguishable from each other.

	Δ Threshold (dB)			Δ Slope (dB)		
(x,y)	95% CI (lower)	95% CI (upper)	*p* value	95% CI (lower)	95% CI (upper)	*p* value
3°,3°	−0.71	+0.29	0.45	−0.64	+0.56	0.85
9°,9°	−0.68	+0.34	0.53	−0.79	+0.53	0.82
15°,15°	−0.85	+0.53	0.58	−0.84	+0.98	0.79
27°,3°	−0.87	+0.14	0.20	−0.45	+0.86	0.47

**Figure 5.**
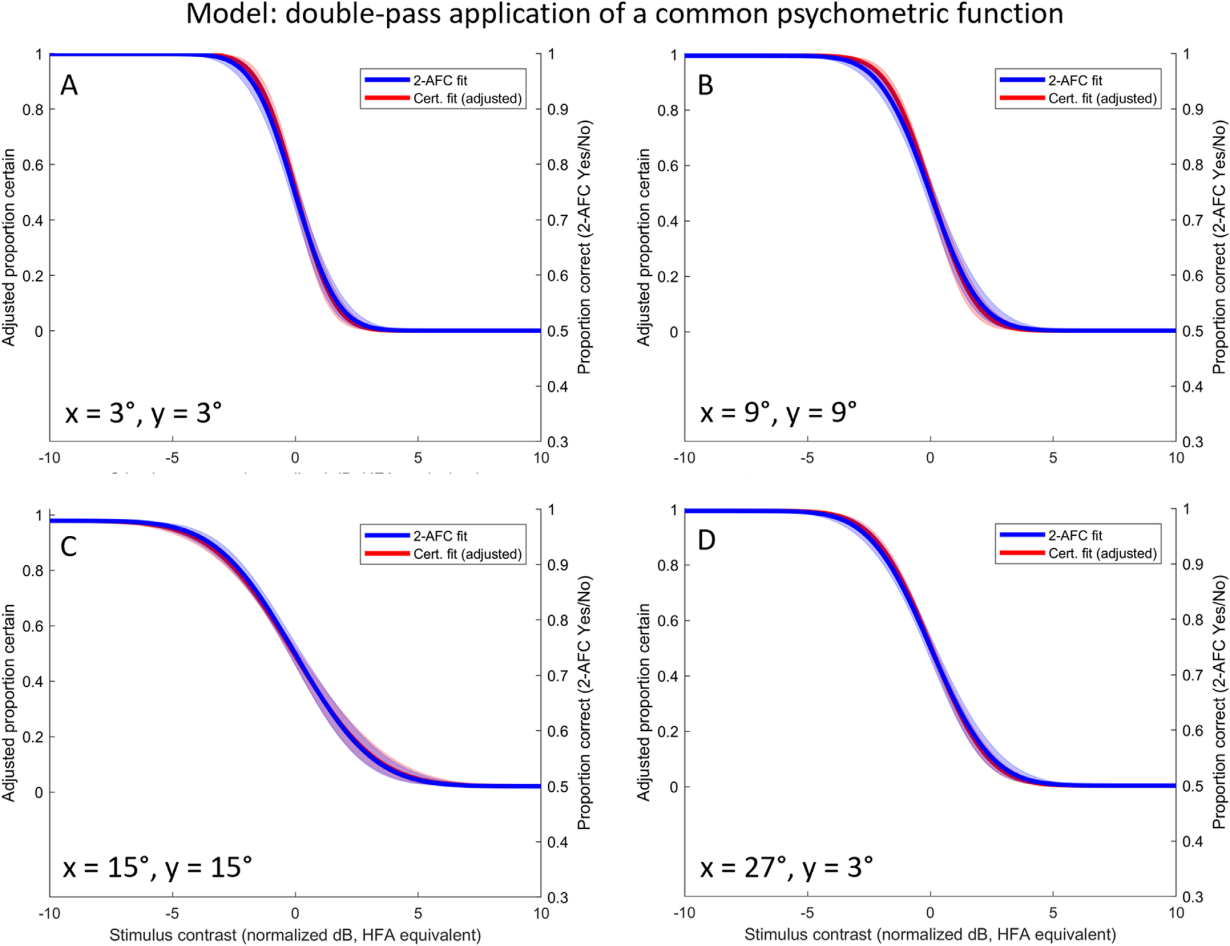
Fitted curve for proportion certain transformed by a square-root operation. This transformation presumes a common psychometric function underlying detection and certainty; applied once to detect the stimulus and, if detected, a second time to determine whether the detection was certain. As for Figures 3 and 4, lines show fitted curves and shaded regions show 95% confidence intervals, with *blue* indicating detection and *red* indicating certainty. Individual points were omitted because the applied transformation can produce non-sensical (negative) values for probability of certainty. For all eccentricities the fitted threshold and slope parameters are indistinguishable under the transformation applied (visual comparison of confidence intervals; also see Table 3).

Given the empirical similarity between functions for detection and for certainty (given correct), it may be possible to predict the threshold for detection from the certainty data alone. We considered a two-stage model whereby a single underlying psychometric function is first sampled to determine whether a stimulus is detected and then, if so, sampled a second time to determine whether it is detected with certainty. To implement this on our data, we fit a curve to the square-root of the proportion certain data, and plotted it against the (non-adjusted) data for proportion correct. The results are shown in Figure 5, and the statistical comparison of fitted parameters in Table 3. Again the two curves appear to be indistinguishable, indicating that detection threshold can be predicted from certainty data (to within the precision afforded by our experiment).

**Table 3.** Difference in fitted parameters between proportion correct (2-AFC) and adjusted certainty data (square-root of proportion certain). For all four eccentricities tested there were no measurable differences in either threshold or slope, meaning that the underlying curves were not distinguishable from each other.

	Δ Threshold (dB)			Δ Slope (dB)		
(x,y)	95% CI (lower)	95% CI (upper)	*p* value	95% CI (lower)	95% CI (upper)	*p* value
3°,3°	−0.35	+0.46	0.73	−0.69	+0.42	0.57
9°,9°	−0.39	+0.48	0.77	−0.86	+0.44	0.53
15°,15°	−0.58	+0.57	0.95	−0.79	+0.95	0.79
27°,3°	−0.44	+0.41	0.88	−0.79	+0.37	0.64

### Part II—Simulations (ZEST)

To assess the potential benefit of incorporating certainty information in an adaptive testing strategy for efficient estimation of threshold, we performed Monte Carlo simulations of a large number of individual ZEST runs for a Yes/No detection task, with or without augmentation from a concurrent Certain/Uncertain task. These simulations sampled a common underlying psychometric function for both detection and certainty-given-detection, following our above finding that there appears to be little discernible difference between these functions. Our approach also presumes that this parity, established for 2-AFC, remains true under a Yes/No paradigm.

Three Bayesian-inspired (ZEST) strategies were assessed, as detailed in the Methods and Figure 2:

i) Conventional ZEST: Simple Yes/No, ignoring certainty information.
ii) Independent PDFs: Yes/No and Certain/Uncertain functions had independent probability distributions; wherever a threshold estimate was required, this was determined from each function independently and then an average taken.
iii) Combined PDF: Yes/No and Certain/Uncertain functions were combined in a single probability distribution.

**Figure 6.**
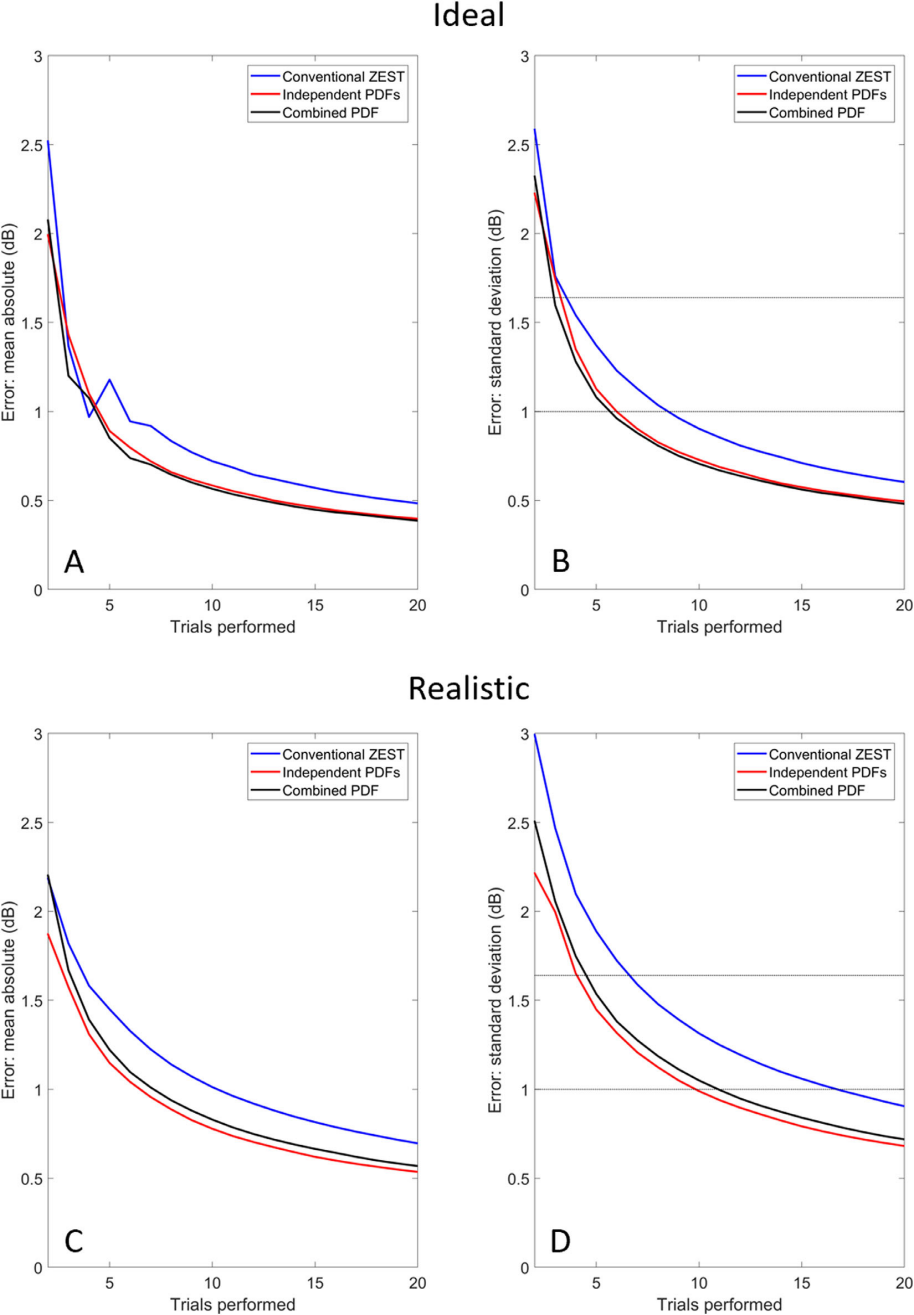
Simulated ZEST performance in a detection task with and without the use of certainty information. Top row shows an “ideal” scenario in which the presumed threshold and slope were equal to the actual threshold and slope, and no response errors were made. (A) Shows the mean absolute error from 10,000 simulations, with a conventional ZEST (Yes/No) approach plotted in *blue*, the “independent PDF” approach in *red*, and the “combined PDF” approach in *black*. (B) As for (A), but plotting the standard deviation of error across the 10,000 simulations. *Horizontal lines* indicate criteria for test-retest reliability (see text). *Bottom row* shows a more “realistic” scenario in which the presumed threshold is in error by 5 dB, the true slope is 1 dB flatter than expected, and response errors are 5%. (C, D) Plot the mean absolute error and standard deviation of error for the “realistic” scenario; plots otherwise as described above for panels (A) and (B).

Figure 6 (top row) shows the predicted performance in an idealised scenario where the initial guess is equal to the true threshold, the presumed slope of the psychometric function is correct, and no response errors of any kind are made. Figure 6A shows mean absolute error as a function of trial number, whereas Figure 6B shows the standard deviation of (signed) error.

Test-retest standard deviation for the Humphrey Field Analyzer's SITA-Standard 24-2 test is approximately 1.64 dB in healthy eyes (Gardiner, 2018); this value is shown as the upper horizontal line in Figure 6B. Approximately 3.5 trials were required to meet this criterion for conventional testing, and 2.9 to 3.3 trials when incorporating certainty information. Differences were more pronounced where the desired precision was higher: for a 1 dB test-retest criterion (lower horizontal line), the number of trials required was approximately 8.5 for conventional testing and 5.7 to 6.0 when incorporating certainty information, thus reducing trials required by 30% to 33%.

The modeling exercise was repeated in the presence of various “realistic” sources of error: actual threshold was 5 dB different from the initial threshold guess, true slope (3 dB) was 1 dB flatter than the presumed slope, and response errors were 5%. The results are shown in Figure 6 (bottom row). As expected, performance was worse in the presence of error for all three methods. However, the benefit of including certainty information was increased. At the 1.64 dB error criterion (upper horizontal line in Figure 6D), conventional testing required 6.6 trials on average, compared with 4.1 to 4.5 with certainty information (reducing trials required by 32% to 39%). For the 1 dB criterion, approximately 16.7 trials were required for conventional testing, compared with 9.8 to 10.9 with certainty. These figures correspond to a saving of trials required of 35% to 41%.

## Discussion

### Overall

In a simple perimetry-style task, our data did not support a measurable difference between the psychometric functions for detection (2-AFC) and for certainty *given* detection. In other words, at visual threshold (defined as 50% chance of detection), when the stimulus was seen, our subjects were certain that they saw it approximately 50% of the time. This was a surprising result, contravening our expectation that one would be somewhere in the 80-95% range on the frequency-of-seeing curve if certain of seeing the stimulus.

When trained observers participate in a typical psychophysical experiment, they are inundated with stimuli that they are *certain* they have seen, which causes the impression that one is being tested inefficiently because such stimuli must surely be well above their threshold (Klein, 2001). The present findings would seem to be at odds with that position, showing that even at threshold one should expect a large proportion of “seen” responses to be seen with high conviction.

Despite contravening our expectations regarding the relationship between performance and certainty, their apparent equality is optimal in regard to using certainty information to more efficiently estimate the shape of the psychometric function for performance. If the two functions can indeed be directly equated, this obviates any need to determine what threshold an observer applies to their internal degree of confidence in order to describe their percept as “certain” (i.e., no additional bias is introduced). In cases where the two functions differ the observer can be said to be either over- or under-confident, and hence some degree of “calibration” would be required to avoid errors in estimation of performance threshold based on Certain/Uncertain data (for further discussion, see “Limitations and further work” below).

Under the assumption that the functions can indeed be equated, our model proposed that the subject decides whether they are certain only if the stimulus has been detected. In this case, one “rolls the dice” again to decide whether the stimulus was detected with certainty; for these trials there is therefore no redundancy between the detection and certainty data. On the other hand if the stimulus is not detected, the certainty response is practically guaranteed to be negative, yielding complete redundancy for this subset of responses. Hence, we expect twice as much information to be obtained, but only on half of the trials, whilst the other half yield the normal amount of information. This means that the expected “value” of each trial, under this model, is 1.5 times that of a normal trial, leading to 2/3 of the number of trials being required. This gain in efficiency may be tempered somewhat by the increased number of responses available; practical strategies to efficiently gather the additional information are discussed further below.

Monte Carlo simulations of adaptive Yes/No testing predicted a reduction in number of trials required to estimate threshold by 30% to 40%, in line with the above prediction. Improvement was especially apparent when considering the variance in returned thresholds (Figure 6B and 6D) and in the presence of error (Figure 6C and 6D). Decision confidence provides a complementary source of information which may help to “rescue” runs affected by an unlikely string of responses (Kaernbach, 2001). Such improvements would be expected to be even more apparent under a 2-AFC detection paradigm, which is particularly prone to sequences of “lucky guesses.” Thus the proposed use of certainty information has merit both in clinical settings (which may use a Yes/No approach for efficiency) and in basic visual psychophysics (which often use a 2-AFC approach to minimize bias). Although not modeled here, gains in efficiency should be most pronounced when the uncertainty in the threshold estimate is high, i.e., when the slope of the psychometric function is shallow (King-Smith et al., 1994). Particularly shallow slopes are observed in moderate perimetric loss in glaucoma (thresholds below approximately 19 dB), resulting in a general inability to reliably determine threshold within a conventional number of trials (Gardiner, Swanson, Goren, Mansberger, & Demirel, 2014).

The improvement in efficiency predicted with the use of certainty information, as expressed by the proportion of trials saved, was relatively constant for different criteria and for different overall numbers of trials. Again this suggests that the method may have merit both for basic science applications, where subjects are typically “well-trained” and there are many trials, and in clinical settings where they are not well trained and there are fewer trials. The predicted degree of improvement was around 30% fewer trials required in the no-error condition, and around 40% in the error-prone condition. This corresponded (Figure 6) to a reduction in variability of around 20% to 30%, respectively. This compares favorably with previous modeling predicting that reductions in variability of 20% or more would make a clinically meaningful improvement to the detection of progression in glaucoma (Turpin & McKendrick, 2011).

An important consideration in efficient adaptive psychometric testing is the selection of starting intensity. It has been proposed that in Yes/No paradigms, presenting real observers with stimuli near threshold which do not happen to be seen may cause them to “panic”, shifting their response criterion to be more lax. This would lead to bias in returned threshold estimates and a higher false positive rate (Heijl et al., 2019; Phu, Khuu, et al., 2019). A similar “panic” response can occur for unlucky sequences of non-seen stimuli beyond the initial phase, where the observer has not pressed the button for some time and feels obligated to do so. Such problems could be ameliorated by the use of certainty information, using a strategy for initial testing that seeks to obtain at least one “certain” response to build the confidence of the participant that they are performing the task correctly. Although such subjective nuances are outside the scope of the simple resilience to error evaluated here, they should be considered in future practical implementations.

Our predictions for the degree of efficiency gain with use of certainty information are in broad agreement with those of Lesmes et al (2015), who presented a signal detection theory framework incorporating the extra response “Uncertain” to a Yes/No detection task (Lesmes et al., 2015). The choices of Yes, No, and Uncertain may effectively promote the “Yes” response to the level of “Certain” (or at least, “Not Uncertain”); thus their rating task appears analogous to ours. They reported that variability of 1 dB was reached after approximately 25 trials for a Yes/No task, which dropped to 15 trials with the addition of the extra response option. This is a reduction of 40% in number of trials required, in line with our predictions for the “Realistic” scenario modelled in Figure 6D. The total number of trials was somewhat higher for their approach (around 50% higher), most likely due to the large number of catch trials used to optimize performance according to signal detection theory.

The typical response apparatus in clinical perimetry is designed for maximum simplicity: subjects are given a single button to press when they see a spot (“Yes”), and a lack of button-press is presumed to indicate a “No.” To collect certainty information in such a setting, more patient input is required. This could be accomplished more economically than the four-button approach used here, which may be troublesome for elderly or inexperienced observers given the potentially increased cognitive load of the task. This could be accomplished by provision of an additional button (requiring a hardware change), a double-click of a single button (no hardware change, but potentially difficult for some subjects), or holding down the button for an extended period instead of a quick click (currently used as a subject-initiated pause in many clinical instruments).

The response paradigm just described for clinical perimetry mandates the use of a finite response period, with a lack of response within the period indicating “No.” This may “interrupt” subjects who were taking a little longer to contemplate a noisy percept, but would have responded “Yes” if given more time (indeed, previous work has advocated the use of reaction time as a surrogate for response confidence as discussed further below). This interruption would tend to elevate their threshold measured for detection. In comparison, the threshold for certainty would be largely unaffected because subjects tend to respond quickly when they are certain. The elevation of threshold for detection, although certainty remains constant, would tend to reduce the distance between the two measured thresholds. This should increase the degree to which one may be used as a proxy for the other.

The best method for combining certainty and detection data is unknown. We modelled two possibilities: one which maintained two parallel probability functions (one for detection and one for certainty) and took an average of the two estimates when choosing the next trial and deciding upon the final threshold; and the other which maintained a single probability function which was multiplied by multiple likelihood functions where appropriate. Our simulations predict these methods to be largely equivalent under “ideal” conditions (no response errors, correctly guessed threshold and slope). Under “realistic” conditions with moderate levels of error, the “independent PDF” approach required fewer trials for a given level of accuracy. Considering isolated errors, the independent PDF approach may be more resilient because one PDF remains unpolluted by the error.

### Limitations and further work

Our observers were young and also well-trained after undergoing multiple MOCS runs that each consisted of 280 trials. Inexperienced and elderly observers tend to show a greater divide between their self-confidence and their performance, with the typical finding in simple perceptual tasks (such as this one) being one of “underconfidence” (Björkman, Juslin, & Winman, 1993; Keren, 1991). Typically this would be taken to mean that the threshold for achieving a confidence rating of 50%, on a scale of 0% to 100%, would be higher than the performance threshold (for example, 50% hit rate on a Yes/No detection task). It is not fully understood how a binary classification of “absolutely certain” as considered here should map onto a confidence rating scale (e.g., whether the stimulus intensity giving 50% of responses as certain would correspond to an average confidence rating of 50% on a scale of 0%–100%). If one considers a simple mapping whereby the probability of being certain at a given intensity is equal to the average confidence rating on a scale of 0% to 100%, our data would also support underconfidence because thresholds were around 0.5 to 1 dB worse for certainty (Figure 3, Table 1).

However, we have proposed that it is sensible to “correct” certainty data for non-detected trials and, at least in our young and reasonably well-trained observers, this caused their thresholds for detection and for certainty (given detection) to appear indistinguishable. Regardless of this correction, given the importance in day-to-day life of achieving a high level of certainty in visual perceptual tasks (such as crossing a busy road), measures of *certainty* may more closely mirror performance than ratings of confidence. It remains to be explored how well performance and certainty are coupled in elderly or inexperienced subjects, especially given the potentially increased cognitive load of the task), and it is also worth noting that any such finding will need to be established on a task-specific basis; for example it is known that more difficult tasks can be associated with overconfidence (Baranski & Petrusic, 1994), in a complex manner that depends on both accumulated evidence and the time required to reach a decision (Kiani, Corthell, & Shadlen, 2014).

Reaction time has previously been proposed as a surrogate for distance from threshold in clinical perimetry, and, hence, as a proxy for response confidence (McKendrick et al., 2014; Wall, Kutzko, & Chauhan, 2002). The separability of the time taken to reach a decision from the evidence actually available to make the decision (Kiani et al., 2014) places an upper bound on the utility of such an approach. However, in cases that do benefit from the use of reaction time data, the incremental utility of using confidence data may be limited. Future work should consider, on an application-specific basis, the relative merits of response time and confidence ratings (or ratings of certainty, as we have argued may be more robust).

Differences between the psychometric functions for detection and for certainty could reduce the gains in efficiency predicted by our Monte Carlo modelling. This is relevant given the specific application that we have considered (detection perimetry). The relationship between detection and certainty could be influenced by a host of factors not explored here. For example, perimetry typically involves splitting of one's attention across the visual field. This has been shown to reduce sensitivity, possibly due to a reduction in certainty, with a more pronounced difference being reported in pathological cases (Phu et al., 2018). It will be of interest to learn how well conserved the correspondence between detection and certainty, observed here, is when extended to a diverse array of visual functions and testing conditions and in the presence of age- or disease-related changes to the visual system.

Detection perimetry is often performed on elderly and/or inexperienced observers. Such tests are also performed with far fewer presentations (approximately two to six trials (Bengtsson & Heijl, 1998)) than we have used here (280 trials) due to time and attention constraints in clinical evaluation of a large number of points across the visual field. Accordingly, the typical test-retest standard deviation is relatively high in such tests, e.g., approximately 1.6 dB for the Humphrey Field Analyzer SITA-Standard 24-2 test (Gardiner et al., 2014). For comparison, our “uncorrected” thresholds for a certain response were 0.5 to 1.0 dB worse that detection thresholds, i.e., within the test-retest error of clinical perimetry. Thus there is good reason to hope that in clinical settings, the approximate equivalence between detection and certainty measures will hold to within measurable limits.

A further potential limitation in clinical populations is that for a disease such as glaucoma in which neural machinery is damaged, the normal coupling between performance and certainty could break down. The visual system could plausibly be more “confused” by received stimuli due to misfiring and missing cells, limiting the ability to use certainty information to rapidly infer detection threshold. However, if such putative differences were indeed observed, they could provide an additional source of diagnostic information. Alternatively, given that the primary site of damage in glaucoma is held to be within the eye whereas the neural machinery underlying certainty is predominantly cortical (Fleming, Weil, Nagy, Dolan, & Rees, 2010; Kiani & Shadlen, 2009), certainty thresholds may be found to simply shift in line with detection thresholds. Of course, confidence measures could be dissociated from performance where the cortex is “damaged” (independently of the retina), as could occur for example in aging or dementia (Grimaldi, Lau, & Basso, 2015). Again, if significant then such a limitation could actually provide useful diagnostic information if an appropriate baseline had been established.

The empirical data presented in Figures 3, 4, and 5 compared thresholds for certainty and performance on a 2-AFC task, but the modeling in Figure 6 considered a Yes/No task that is more relevant to clinical perimetry. The relationship between performance and certainty could well differ when using a Yes/No paradigm; when not forced to guess, untrained subjects will adopt varying response criteria that are expected to produce elevated thresholds. It is not presently known whether the threshold for Certain/Uncertain would shift accordingly, or whether it might in fact remain relatively stable despite shifts in decision criteria for detection. In other words, perhaps criterion variability is less when subjects are asked whether they were “absolutely certain,” as opposed to just whether they saw the stimulus or not.

In classical theories of decision making based on signal detection theory (SDT), degree of confidence is modelled by the distance between the stimulus intensity and the internal decision criterion (Kepecs, Uchida, Zariwala, & Mainen, 2008). For those trials that turned out to be incorrect, SDT predicts decreasing confidence with increasing stimulus intensity; recent evidence suggests that the opposite is observed in practice (Kiani et al., 2014). Here we have proposed a third possibility when confidence is expressed as a binary classification of “absolutely certain” or not: subjects do not (other than mis-clicks) report being “absolutely certain” when they have not in fact seen the stimulus. This is reconcilable with SDT because when it comes to a binary classification of certainty, subjects are not in fact “detecting” a stimulus, rather they are making a comparison of the degree of confidence elicited by the stimulus to their internal register of what should constitute sufficient confidence to be labeled “absolutely certain.” In other words, the task has been transformed into a discrimination task, for which a zero false alarm rate is not inconsistent with SDT (Klein, 2001). This is one way in which there may be a meaningful difference between confidence ratings and binary classification of Certain/Uncertain, suggesting the latter as a useful avenue of future investigation in the field of perceptual decision making.

## Conclusions

In young, healthy subjects the probability of spot detection across the visual field was approximately equal to the probability of being certain, given detection. This equivalence could allow threshold and slope of the psychometric function for detection to be estimated from certainty responses. Simulations based on this relationship predict a significant reduction in the number of trials required to estimate detection threshold, especially within clinically relevant margins of accuracy.
